# Significance of fragmented QRS complexes for identifying culprit lesions in patients with non-ST-elevation myocardial infarction: a single-center, retrospective analysis of 183 cases

**DOI:** 10.1186/1471-2261-12-44

**Published:** 2012-06-19

**Authors:** Rong Guo, Yuanmin Li, Yawei Xu, Kai Tang, Weimin Li

**Affiliations:** 1 Department of Cardiology, Shanghai Tenth People's Hospital, Tongji University School of Medicine, Shanghai, 200072, China

**Keywords:** Fragmented QRS complexes, Coronary artery disease, Acute coronary syndrome, Electrocardiogram

## Abstract

**Background:**

Fragmented QRS (fQRS) complexes are novel electrocardiographic signals, which reflect myocardial conduction delays in patients with coronary artery disease (CAD). The importance of fQRS complexes in identifying culprit vessels was evaluated in this retrospective study.

**Methods:**

A 12-lead surface electrocardiogram was obtained in 183 patients who had non-ST-elevation myocardial infarction (NSTEMI) and subsequently underwent coronary angiography (CAG). On the basis of the frequency of fQRS complexes, indices such as sensitivity, specificity, positive and negative predictive values, and likelihood ratio were evaluated to determine the ability of fQRS complexes to identify the culprit vessels.

**Results:**

Among the patients studied, elderly patients (age ≥ 65 years) and those with diabetes had a significantly higher frequency of fQRS complexes (*p* = 0.005, *p* = 0.003, respectively). The fQRS complexes recorded in the 4 precordial leads had the highest specificity (81.8%) for indentifying the culprit vessel (left anterior descending artery). However, the specificity of fQRS complexes to identify lesions in the left circumflex and right coronary arteries was lower for the inferior and lateral leads than for the limb leads (65.5% versus 71.7%); however, the limb leads had higher sensitivity (92.3% versus 89.4%). And the total sensitivity and specificity of fQRS (77.1% and 71.5%) were higher than those values for ischemic T-waves.

**Conclusions:**

The frequency of fQRS complexes was higher in elderly and diabetic patients with NSTEMI. The frequency of fQRS complexes recorded in each of the ECG leads can be used to identify culprit vessels in patients with NSTEMI.

## Background

The diagnosis of non-ST-elevation myocardial infarction (NSTEMI) depends on a thorough physical examination, detailed clinical history, and immediate interpretation of the results of a resting 12-lead echocardiogram (ECG). Therefore, the ECG can provide crucial information for diagnosing NSTEMI. Other ECG abnormalities such as T-wave inversion, ST-segment depression, microvolt T-wave alternans, late potentials on the signal-averaged ECG, and pathologic Q waves have diagnostic value, but their correlation with the exact anatomic location of the culprit lesion is not very high.

Fragmented QRS (fQRS) complexes are novel electrocardiographic signals, which reflect altered ventricular conduction delays around regions of a myocardial scar. The detection of fQRS complexes in a routine 12-lead ECG is a marker for abnormal cardiac depolarization. Previous studies have shown that fQRS complexes are useful for diagnosing coronary artery disease (CAD) [1,2]. However, limited information is available on the diagnostic utility of fQRS complexes in patients with NSTEMI. In this study, we reviewed the ECG results in NSTEMI patients to evaluate the accuracy of fQRS complexes to identify culprit lesions.

## Methods

### Study population

From January 2010 to April 2011, we conducted a retrospective study on 183 consecutive patients diagnosed with NSTEMI in our department. Standard 12-lead ECGs, data on cardiac biomarkers, and echocardiographic and coronary angiography (CAG) findings were collected from the study subjects. The patient demographics, including history of significant CAD (prior myocardial infarction or typical angina pectoris), previous percutaneous coronary intervention (PCI) or coronary artery bypass grafting (CABG), and risk factors for CAD (age, sex, hypertension, smoking, diabetes mellitus or hyperlipidemia) were noted.

Patients were eligible for inclusion in the study if they had a confirmed NSTEMI and an fQR**S** complex in any of the ECG leads recorded during the NSTEMI episode. Patients with permanent atrial fibrillation, ventricular paced rhythm, a previously implanted implantable cardioverter-defibrillator (ICD) or a clinical indication for an ICD at the time of enrollment, left ventricular hypertrophy, Wolff-Parkinson-White syndrome, cardiomyopathy, myocarditis, and congenital heart disease were excluded from the study. NSTEMI was defined as ST-segment depression or prominent T-wave inversion and/or positive tests for biomarkers of necrosis in the absence of ST-segment elevation detected in an appropriate clinical setting (i.e., when the patient experienced chest discomfort or angina) [3].

This study was approved by the institutional ethics committee, and informed consent was obtained from all enrolled patients.

### ECG

The ECG and supplemental criteria for fQRS patterns were defined by Das [1,4]. The resting 12-lead ECG (filter range, 0.15–100 Hz; AC filter, 60 Hz, 25 mm/s, 10 mm/mV) was analyzed by 2 independent readers blinded to the CAG results. The fQRS pattern was defined as the presence of an additional R’ or crochetage wave, notching in the nadir of the S wave or fragmentation of the RS or QS complexes in 2 contiguous leads corresponding to a major coronary artery territory. The fQRS pattern could occur in patients with or without Q waves. However, patients with a typical bundle-branch block pattern (QRS ≥ 120 ms) or incomplete right bundle-branch block pattern were excluded. Furthermore, patients with a pathological Q wave in the ECG and a history of prior myocardial infarction were also excluded.

### CAG

The severity of stenoses was graded using the Coronary Angiogram Analyzing System II (CAAS II; Pie Medical, Maastricht, The Netherlands)[5]. Coronary flow over the culprit lesion was graded according to the Thrombolysis in Myocardial Infarction Trial (TIMI) criteria, and collateral circulation was classified according to the criteria proposed by Rentrop et al [6,7]. Multivessel CAD was defined as the presence of lesions in 3 or more coronary vessels. The presence of occlusion in the main and secondary branch of a vessel was defined as single-vessel disease.

CAG was performed in all 183 patients included in the study. Of these patients, 87 underwent angiography during the first 24 h of the NSTEMI episode, and 96 underwent CAG within 2 to 14 days after the acute episode. The location of coronary lesions, the number of stenosed arteries and the degree of stenosis were recorded for each patient.

### Statistical analysis

Values are expressed as mean ± standard deviation. A two-tailed Student’s *t*-test was used for comparing continuous variables. A Chi-square test or Fisher’s exact test was used to compare dichotomous data. To evaluate the diagnostic value of fQRS complexes in each patient, indices such as sensitivity, specificity, likelihood ratio, and positive and negative predictive values were recorded. Logistic regression analysis was used to examine the influence of various clinical factors by estimating the probability of fQRS occurrence on an ECG. Receiver operating characteristic (ROC) curves were used to assess the relationship between fQRS complexes and ischemic T-waves for the diagnosis of NSTEMI. SPSS 13.0 was used for all statistical analyses. A *p* value < 0.05 was considered statistically significant.

## Results

### Demographics and clinical data

The ECG findings of the patients showed that fQRS complexes were present in 60.1% of all patients with NSTEMI (n = 183). The remaining 73 patients with ST-segment depression or prominent T-wave inversion formed the control group. Advanced age and the prevalence of diabetes were higher in the fQRS group (*p* < 0.05). No significant differences were observed in renal function between the two groups (Table 1). Logistic regression analysis showed that old age (≥ 65 years), cardiac troponin T (cTnT) levels and diabetes were significantly associated with the presence of fQRS complexes. The odds ratios (OR) for old age, cTnT and diabetes were 2.04 (95% CI, 1.09–3.09; *p* = 0.026), 0.73 (95% CI, 0.55–0.98; *p* = 0.036) and 2.05 (95% CI, 1.06–3.97; *p* = 0.033), respectively (Table 2).

**Table 1:** Baseline characteristics of enrolled patients

	**fQRS group (n = 110)**	**Non-fQRS group (n = 73)**	***p*-value**
Age (yrs)	64 ± 1.0	59 ± 1.0	0.005
Gender (M/F)	70/40	47/26	0.918
Hypertension	70.9% (78)	63.0% (46)	0.263
Hyperlipidemia	61.8% (68)	67.1% (49)	0.464
Diabetes mellitus	73.6% (81)	52.1% (38)	0.003
Tobacco use	58.2% (64)	60.3% (44)	0.778
cTnT (ng/ml)	1.35 ± 0.12	1.03 ± 0.10	0.049
Prior PCI	41.8% (46)	46.6% (34)	0.525
Prior CABG	11.8% (13)	12.3% (9)	0.917
LVEF (%)	57.3 ± 10.3	57.1 ± 11.1	0.933
Cockcroft-Gault formula (CG-GFR) (ml/min)	81.16 ± 15.67	83.52 ± 15.41	0.299

M = males; F = females; cTnT = cardiac Troponin T; PCI = percutaneous coronary intervention; CABG = coronary artery bypass grafting; LVEF = left ventricular ejection fraction; Cockcroft-Gault formula (CG-GFR) was used to estimate renal function.

**Table 2:** Logistic regression analysis

	***p*-value**	**OR**	**95% CI**
Hypertension	0.327	1.401	0.71–2.75
Hyperlipidemia	0.824	0.928	0.48–1.80
Tobacco use	0.565	1.212	0.63–2.33
Diabetes	0.033	2.052	1.06–3.97
cTnT	0.036	0.731	0.55–0.98
Prior PCI	0.973	0.989	0.53–1.86
Prior CABG	0.834	1.106	0.43–2.83
Gender	0.632	0.936	0.48–1.83
LVEF	0.909	0.998	0.97–1.03
Old age (≥ 65 yrs)	0.026	2.042	1.09–3.84

Variable(s): hypertension, hyperlipidemia, tobacco use, diabetes, cardiac troponin T (cTnT), percutaneous coronary intervention (PCI), coronary artery bypass grafting (CABG), gender, left ventricular ejection fraction (LVEF), old age.

### CAG

Out of the 183 patients, 42 showed left coronary artery dominance, 125 showed right dominance and 16 had a balanced coronary system. The incidence of triple-vessel disease was higher in the fQRS group than that in the control group (*p* = 0.002). The incidence of 3-vessels disease were quite higher in fQRS group (p = 0.002). Similarly, severe and mild degree of coronary stenosis in fQRS group were much higher than that of non-fQRS group (p = 0.038; p = 0.001) (Table 3).

**Table 3:** Comparison of CAG results between the 2 groups

	**fQRS group (n = 110)**	**Non-fQRS group (n = 73)**	***p*-value**
Coronary Lesions			
Single-vessel disease	23	14	0.775
Double-vessel disease	38	43	0.193
Triple-vessel disease	49	16	0.002
Culprit vessel			
LAD	102	63	0.830
LCX	63	37	0.893
RCA	81	48	0.919
Degree of coronary stenosis			
90% < D < 100%	54	20	0.038
70% < D ≤ 90%	125	62	0.086
50% ≤ D ≤ 70%	67	66	0.001

LAD = left anterior descending artery; LCX = left circumflex artery; RCA = right coronary artery; D = diameter.

### The diagnostic importance of fQRS complexes in the 12-lead ECG

The frequency of fQRS recorded in each ECG lead was related to the culprit vessel or lesion in patients with NSTEMI. The sensitivity of fQRS in 2 anterior ECG leads was the highest (80.9%), but the specificity was only 68.4%. The specificity of fQRS in 4 anterior ECG leads was the highest (81.8%), but the sensitivity was only 62.7%.

The sensitivity, specificity, and positive and negative predictive values of fQRS in ECG leads II, III, and aVF were 92.3%, 65.5%, 85.6, and 79.2%, respectively; the sensitivity, specificity, and positive and negative predictive values of fQRS in ECG leads I, aVL, and V6 were 89.4%, 71.7%, 83.5, and 80.9%, respectively. Our results confirmed that the specificity of fQRS complexes in identifying lesions in the left circumflex and right coronary arteries was lower for the inferior and lateral leads than that for the limb leads (65.5% versus 71.7%); however, the former had higher sensitivity (92.3% versus 89.4%) (Table 4).

**Table 4:** Electrocardiographic predictors of culprit lesions

	**Predictors of LAD lesion**					
	**Sens**	**Spec**	**PPV**	**NPV**	**LR (+)**	**LR (−)**
fQRS in 2 anterior leads	80.9%	68.4%	77.1%	57.7%	1.99	0.54
fQRS in 3 anterior leads	73.0%	79.8%	85.8%	63.9%	3.61	0.34
fQRS in 4 anterior leads	62.7%	81.8%	93.3%	52.0%	4.45	0.23
fQRS in 5 anterior leads	38.6%	75.7%	68.4%	47.6%	1.59	0.81
	Predictors of RCA lesion					
	Sens	Spec	PPV	NPV	LR (+)	LR (−)
fQRS in inferior leads	92.3%	65.5%	85.6%	79.2%	2.68	0.12
	Predictors of LCX lesion					
	Sens	Spec	PPV	NPV	LR (+)	LR (−)
fQRS in I, aVL, and V6 leads	89.4%	71.7%	83.5%	80.9%	3.16	0.15

LAD = left anterior descending artery; RCA = right coronary artery; LCX = left circumflex artery; Sens = sensitivity; Spec = specificity; PPV = positive predictive value; NPV = negative predictive value; LR = likelihood ratio.

### Comparison of the diagnostic accuracy between fQRS and ischemic T-waves

The presence of fQRS for the diagnosis of left anterior artery (LAD) lesions was less sensitive (58.0% versus 62.1%) but more specific (75.00% versus 58.2%) compared with the presence of ischemic T-waves. The sensitivity and specificity of fQRS for the diagnosis of left circumflex artery (LCx) lesions were 89.4% and 71.7% compared with 53.4% and 70.6% for ischemic T-waves, respectively. For the diagnosis of right coronary artery (RCA) lesions, the presence of fQRS was more sensitive (92.3% versus 66.2%) and less specific (65.5% versus 66.3%) than ischemic T-waves. We found that the total sensitivity and specificity of fQRS (77.1% and 71.5%) were higher than those values for ischemic T-waves.

**Figure 1:**
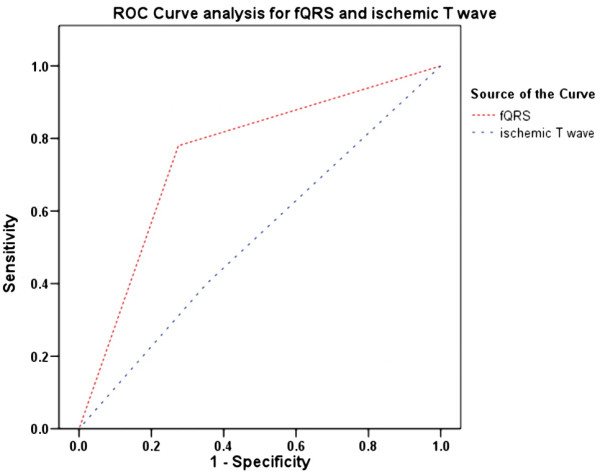
ROC curve analysis to determine the accuracy of fQRS complexes and ischemic T-waves to diagnose NSTEMI.

**Figure 2:**
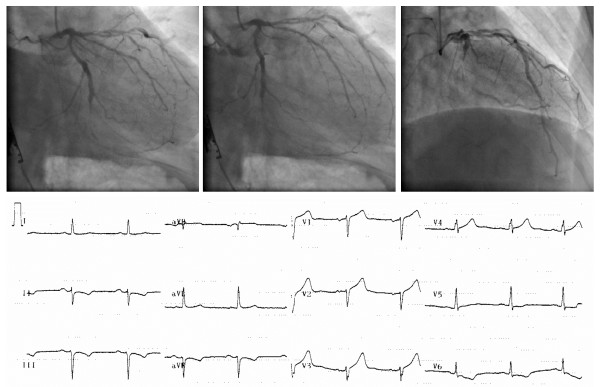
**A patient’s CAG image showing severe diffusive atherosclerosis. The middle part of the LCX was totally occluded. Several atherosclerotic plaques and narrowings can be seen in the LAD.** The fQRS complexes were observed in the V2–V4 precordial ECG leads.

Receiver operating characteristic (ROC) curves were used to evaluate the diagnostic accuracy of fQRS and ischemic T-waves for the diagnosis of culprit lesions in patients with NSTEMI. The areas under the ROC curves for fQRS and ischemic T-waves were 0.75 (95% CI, 0.66–0.85) and 0.54 (95% CI, 0.41–0.64), respectively. Thus, the total diagnostic accuracy was significantly higher for fQRS than that for ischemic T-waves (Figure 1 and 2; *p* = 0.03).

## Discussion

### Clinical risk factors

In patients with NSTEMI, only a part of the artery is occluded. This implies that only a portion of cardiomyocytes supplied by the occluded artery are necrotic. In the NSTEMI patients in the fQRS group, fQRS complexes were significantly more common in older patients (≥ 65 years) and those with diabetes (OR, 2.04 *p* = 0.026] and OR, 2.05 *p* = 0.033], respectively). It has been established that the number of risk factors and complications are higher in elderly patients [8,9]. Moreover, a previous study has shown that diabetes can cause extensive vascular damage and increase the number of multivessel lesions [10]; this could explain the higher incidence of fQRS complexes in patients with diabetes.

### ECG analysis

According to the standard distribution of the major coronary arteries in humans, fQRS complexes detected in 2 or more contiguous anterior leads (V1–V5) were assigned to the left anterior descending artery territory. The presence of fQRS complexes in the lateral leads (I, aVL, and V6) were assigned to the left circumflex artery territory, and those in the inferior leads (II, III, and aVF) were assigned to the right coronary artery territory. We analyzed the frequency of fQRS in each lead and compared the frequencies with CAG findings. The frequency of fQRS was higher in the precordial leads than in other leads and showed higher specificity but lower sensitivity for predicting LAD lesions. The presence of fQRS complexes recorded in 4 precordial leads had the highest specificity for the identification of the culprit vessel (81.8%) (Table 4). Although the specificity of the inferior and lateral lead recordings to identify LCX and RCA lesions was low (65.5% versus 71.7%), the sensitivity was considerably higher (92.3% versus 89.4%).

ST-segment depression and/or T-wave inversion in the ECG usually reflects the abnormal repolarization of cardiac tissues caused by myocardial ischemia in patients with CAD. ST-segment elevation is an important ECG criterion for the diagnosis of STEMI, but the sensitivity and specificity of ST-segment depression and/or T-wave inversion for predicting the culprit lesion are not very high. Our results showed that the presence of fQRS complexes had better diagnostic accuracy than ischemic T-waves for the identification of culprit vessels (Figure 1; *p* = 0.03). These findings also suggest that fQRS could identify the correct culprit lesion in some patients without ischemic T-wave changes.

Although the prognostic significance of fragmented QRS complexes in patients who underwent primary PCI was confirmed in previous studies, the diagnostic value of fQRS in patients who have a history of revascularization (PCI or CABG is uncertain [2,4,11]. We did not find a more frequent history of PCI or CABG in the fQRS group (*p* = 0.525; *p* = 0.917). Furthermore, logistic regression analysis did not show that prior PCI or CABG were significantly associated with the presence of fQRS complexes (*p* = 0.973; *p* = 0.834). Therefore, the diagnosis of culprit lesions in NESTEMI patients with a prior history of CAD and revasularization should be based on the combination of dynamic ECG changes including fQRS complexes and other clinical manifestations.

### Potential mechanisms

The important of fQRS complexes was first suggested by Das in 2006. The exact mechanism of fragmentation of the QRS complex in the 12-lead surface ECG is still unclear. Previous studies have shown [12,13] that the post-infarction scar tissue morphology correlated well with the patterns of the fQRS complex. The fQRS complexes have been implicated in the inhomogeneous activation of the ventricles due to myocardial scar and/or ischemia. These studies suggest that different fQRS patterns result from shifting of the QRS vector during depolarization of areas around f scar or ischemic myocardium, depending on the extent of injury and its location in the ventricles [14,15].

## Conclusions

Although there have been reports on the use of the fQRS complex as a marker of acute myocardial infarction and remote myocardial infarction scar, not much is known about the diagnostic significance of fQRS in patients with NSTEMI. We found that fQRS complexes occurred more frequently in elderly and diabetic patients with NSTEMI. The frequencies of fQRS complexes recorded in each ECG lead could identify the culprit vessel, and this was particularly useful when the LAD was the culprit vessel. In addition, the diagnostic accuracy of fQRS complexes was significantly higher than that of ischemic T-waves for the diagnosis of NSTEMI.

### Limitations

There are several limitations of this study. The relatively small sample size limited the diagnostic value in clinical practice. Therefore, additional prospective data are needed in a larger study population to confirm our findings. Furthermore, the time that fQRS complexes developed in the ECG, and the exact mechanisms of fQRS are unknown at the present time. The utility of fQRS for identifying culprit lesions in NSTEMI patients with a history of PCI or CABG needs further exploration.

## Competing interest

The authors declare no conflict of interest.

## Authors’ contributions

WL carried out the whole studies and participated in the patients’ enrollment. YL and KT carried out the analysis and interpretation of ECGs. RG drafted the manuscript, participated in the design of the study and performed the statistical analysis. YX conceived of the study, participated in its design and coordination and helped to draft the manuscript. All authors read and approved the final manuscript.

## Pre-publication history

The pre-publication history for this paper can be accessed here:

http://www.biomedcentral.com/1471-2261/12/44/prepub

## References

[B1] Das MK Khan B Jacob S Kumar A Mahenthiran J Significance of a fragmented QRS complex versus a Q wave in patients with coronary artery disease Circulation 2006 113 2495 2501 10.1161/CIRCULATIONAHA.105.595892 16717150

[B2] Das MK Michael MA Suradi H Peng J Sinha A Shen C Mahenthiran J Kovacs RJ Usefulness of fragmented QRS on a 12-lead electrocardiogram in acute coronary syndrome for predicting mortality Am J Cardiol 2009 104 1631 1637 10.1016/j.amjcard.2009.07.046 19962466

[B3] Kushner FG Hand M Smith SC King SB Anderson JL Antman EM Bailey SR Bates ER Blankenship JC Casey DE 2009 focused updates: ACC/AHA guidelines for the management of patients with ST-elevation myocardial infarction (updating the 2004 guideline and 2007 focused update) and ACC/AHA/SCAI guidelines on percutaneous coronary intervention (updating the 2005 guideline and 2007 focused update) a report of the American College of Cardiology Foundation/American Heart Association Task Force on Practice Guidelines J Am Coll Cardiol 2009 54 2205 2241 10.1016/j.jacc.2009.10.015 19942100

[B4] Das MK Suradi H Maskoun W Michael MA Shen C Peng J Dandamudi G Mahenthiran J Fragmented wide QRS on a 12-lead ECG: a sign of myocardial scar and poor prognosis Circ Arrhythm Electrophysiol 2008 1 258 268 10.1161/CIRCEP.107.763284 19808417

[B5] Haase J Escaned J van Swijndregt EM Ozaki Y Gronenschild E Slager CJ Serruys PW Experimental validation of geometric and densitometric coronary measurements on the new generation Cardiovascular Angiography Analysis System (CAAS II) Cathet Cardiovasc Diagn 1993 30 104 114 10.1002/ccd.1810300205 8221861

[B6] Mueller HS DA Greenberg MA The Thrombolysis in Myocardial Infarction (TIMI) trial. Phase I findings. TIMI Study Group N Engl J Med 1985 312 932 936 4038784 10.1056/NEJM198504043121437

[B7] Rentrop KP Cohen M Blanke H Phillips RA Changes in collateral channel filling immediately after controlled coronary artery occlusion by an angioplasty balloon in human subjects J Am Coll Cardiol 1985 5 587 592 10.1016/S0735-1097(85)80380-6 3156171

[B8] Brooks MM Jones RH Bach RG Chaitman BR Kern MJ Orszulak TA Follmann D Sopko G Blackstone EH Califf RM Predictors of mortality and mortality from cardiac causes in the bypass angioplasty revascularization investigation (BARI) randomized trial and registry For the BARI Investigators. Circulation 2000 101 2682 2689 10.1161/01.cir.101.23.2682 10851204

[B9] Best PJ Lennon R Ting HH Bell MR Rihal CS Holmes DR Berger PB The impact of renal insufficiency on clinical outcomes in patients undergoing percutaneous coronary interventions J Am Coll Cardiol 2002 39 1113 1119 10.1016/S0735-1097(02)01745-X 11923033

[B10] Donahoe SM Stewart GC McCabe CH Mohanavelu S Murphy SA Cannon CP Antman EM Diabetes and mortality following acute coronary syndromes JAMA 2007 298 765 775 10.1001/jama.298.7.765 17699010

[B11] Ari H Cetinkaya S Ari S Koca V Bozat T The prognostic significance of a fragmented QRS complex after primary percutaneous coronary intervention Hear Vessel 2012 27 20 28 10.1007/s00380-011-0121-9 21344317

[B12] Flowers NC Horan LG Thomas JR Tolleson WJ The anatomic basis for high-frequency components in the electrocardiogram Circulation 1969 39 531 539 10.1161/01.CIR.39.4.531 5778254

[B13] Gardner PI Ursell PC Fenoglio JJ Wit AL Electrophysiologic and anatomic basis for fractionated electrograms recorded from healed myocardial infarcts Circulation 1985 72 596 611 10.1161/01.CIR.72.3.596 4017211

[B14] el Sherif N The rsR' pattern in left surface leads in ventricular aneurysm Br Heart J 1970 32 440 448 10.1136/hrt.32.4.440 5433304 PMC487350

[B15] Wiener I Mindich B Pitchon R Fragmented endocardial electrical activity in patients with ventricular tachycardia: a new guide to surgical therapy Am Heart J 1984 107 86 90 10.1016/0002-8703(84)90138-8 6691245

